# Moral Insanity

**Published:** 1857-02

**Authors:** 


### EDITORIAL DEPARTMENT.

---

*Moral Insanity.*—"In a late murder trial at Maidstone, England, the plea of murder-mania was urged by the defence, which Judge Bromwell put down in the following common-sense manner:

"If he understood the meaning of the term 'homicidal mania,' it was that a man entertained the bad desire to kill another and could not control it; and he would only observe that if it were to go forth that any man who killed another would escape the consequences if he established such a fact, it would have a most dangerous effect upon society, and it appeared to him that the object of the law was to check such feelings and to teach those who were base enough to entertain them, that certain punishment would follow if they carried them out. The real questions they had to consider were whether the prisoner knew the nature of the act he was committing, and whether he knew it was a wrong act, and if so it would be their duty to say that he was guilty."

We regret to see that some of our cotemporaries indulge in harsh comment upon the course of Profs. Gilman and Parker, in the Huntington trial. In testifying to Huntington's insanity, there cannot be a doubt that these gentlemen did so from pure motives and honest convictions. While, for ourselves, we dissent widely from their conclusions, and look upon their idea as a dangerous one, we are willing to concede to them a high intelligence and an upright purpose. The question is, itself, one of sufficient difficulty to puzzle strong minds, and the boundary between legal responsibility and insanity must ever vary with individual cases.

We look upon the termination of the Huntington trial as a vindication of sound doctrine, but many other cases will arise where it will be impossible for the medical witness to testify, positively, as to the existence of legal responsibility. We are inclined to look with favor on the recent proposal for an amendment of the law, allowing a jury to bring in a verdict of "guilty, with a recommendation to mercy on account of partial insanity;" or a verdict of "not guilty by reason of entire insanity;" as to leave a power of punishment, in doubtful cases, in the hands of the court.

This proposal has other reasons to justify it. We are wrong in looking upon insanity as a *positive quantity* to be measured like the contents of a jug. A person may be insane to some degree, and still be partially responsible for his acts. It would not be far from right to assume that in some cases the patient is guilty of moral dereliction in becoming insane. A great wrong is done a man. Instead of taking legal redress, or calling to his help the grace of Christian forbearance, he broods over his trouble, nurses it, and *reasons* himself into a conviction that he has a right to take the life of the wrong-doer. And coolly, rationally, he commits a murder.

This is, if not murder, a guilty homicide; and the law should punish such cases. The decision should not be suffered to rest on the guilty or not guilty of a jury of clod-hoppers. It should not be assumed that he is either totally guilty or totally innocent. To an enlightened judgment he is guilty of a crime under extenuating circumstances, and the force of those circumstances should govern the degree of his punishment.

We throw out these thoughts in the hope that they may call out a discussion from competent sources. The public mind vibrates like a pendulum between a mawkish mercy and a cruel severity, and the doctrine of chances, the whim of a jury, daily decide the question of life or death without reference to any principle, but simply with regard to the state of an ever-changing public opinion.

